# Preconception Learned Helplessness as a Cognitive Vulnerability to Lower Maternal Bonding Across the Perinatal Period

**DOI:** 10.3390/jcm15166391

**Published:** 2026-08-18

**Authors:** Moriah Azoulay, Sharon Florentin, Adi Klein, Amit Klein, Neta Elbaz Hemo, Tamuz Weinberger, Noa Yakirevich Amir, Keren Avirame, Tal Sido, Atira Bick, Netta Levin, Inbal Reuveni, Shiri Ben-David

**Affiliations:** 1 Department of Psychology, The Hebrew University of Jerusalem, Jerusalem 9190501, Israel; adi.klein@mail.huji.ac.il (A.K.); tamuz.weinberger@mail.huji.ac.il (T.W.); shiri.ben-david@mail.huji.ac.il (S.B.-D.); 2 Department of Psychology, Hadassah Hebrew University Medical Center, Jerusalem 9112001, Israel; 3 Faculty of Medicine, Tel Aviv University, Tel Aviv 6997801, Israel; sharon.florentin@mail.huji.ac.il (S.F.); inbalr@tlvmc.gov.il (I.R.); 4 Psychiatry Division, Tel Aviv Sourasky Medical Center, Tel Aviv 6423906, Israel; kerenav@tlvmc.gov.il; 5 Department of Psychiatry, Hadassah Hebrew University Medical Center, Jerusalem 9112001, Israel; neta.elbaz@mail.huji.ac.il (N.E.H.); noay@hadassah.org.il (N.Y.A.); 6 Department of Obstetrics and Gynecology, Hadassah Medical Center, Jerusalem 9112001, Israel; amitben@hadassah.org.il; 7 Faculty of Medicine, Hadassah Hebrew University Medical Center, Jerusalem 9112001, Israel; tal.sido@mail.huji.ac.il; 8 Department of Neurology, Hadassah Hebrew University Medical Center, Jerusalem 9112001, Israel; bicka@hadassah.org.il (A.B.); netta@hadassah.org.il (N.L.)

**Keywords:** learned helplessness, maternal bonding, antenatal bonding, postpartum bonding, perinatal mental health, cognitive vulnerability

## Abstract

**Background**: Maternal bonding is a key determinant of maternal and infant well-being, yet little is known about preconception factors that shape its development. Learned helplessness (LH), a stable cognitive vulnerability characterized by perceived uncontrollability and diminished efficacy, may predispose women to poorer maternal bonding. This study examined whether preconception LH predicts antenatal and postpartum bonding and whether this association is mediated by antenatal bonding and prenatal affective symptoms. **Methods**: A sample of 151 euthymic, nulliparous women was followed prospectively from preconception through pregnancy and postpartum, completing questionnaires assessing LH, depressive and anxiety symptoms, and antenatal and postpartum bonding. Analyses used structural equation modeling, linear mixed-effects models, hierarchical regression, and parallel mediation. **Results**: LH showed high stability from preconception to postpartum (*r* = 0.71), with no mean-level change, whereas affective symptoms increased. Antenatal bonding predicted postpartum bonding (β = 0.52, *p* < 0.001). Higher preconception LH predicted lower antenatal bonding (*b* = −0.20, *p* = 0.002) and lower postpartum bonding (*b* = −0.29, *p* < 0.001), beyond affective symptoms in the corresponding period. The LH–postpartum bonding association was partially mediated by antenatal bonding (β = −0.10, *p* = 0.005), but not by prenatal depression or anxiety symptoms. **Conclusions**: Preconception LH reflects a stable cognitive vulnerability linked to poorer maternal bonding. Its association with postpartum bonding was partly mediated by antenatal bonding, but not by prenatal affective symptoms, suggesting that LH’s relevance to postpartum bonding begins when the maternal bond develops during pregnancy. Assessing LH before conception or early in pregnancy may help identify vulnerability to lower bonding, beyond current affective symptoms.

## 1. Introduction

A central developmental process in the perinatal period is the formation of mother–infant bonding [1,2,3]. The term “bonding” refers to the establishment of an emotional, behavioral, cognitive, and neurobiological tie between a mother and her infant [4], beginning with the intention to have a child and continuing through pregnancy and the postpartum period [4,5,6,7]. Bonding is a parent-driven process characterized by enduring, committed, and engaged involvement [4] and is underpinned by the oxytocin and dopamine systems that support motivational behaviors such as social orienting, seeking, and maintaining contact [8,9].

Maternal bonding has important implications for both mother and child. Stronger maternal bonding has been linked to more optimal infant development, including higher attachment quality, easier temperament, and more positive infant mood [3,10]. In addition, stronger bonding is positively associated with maternal psychological well-being and has been found to buffer the adverse effects of stress on maternal mental health [11]. Conversely, postpartum bonding difficulties have been linked to disruptions in the long-term mother–child relationship [7], an elevated risk of child abuse or neglect [12], and more social–emotional and behavioral problems in children’s subsequent development [13]. Estimates suggest that 3–22% of mothers in community samples experience bonding difficulties [14,15,16,17,18,19], with higher rates observed in clinical populations [19,20]. Yet individual differences in bonding remain poorly understood and are thought to arise from a combination of psychological, interpersonal, and biological factors [3]. Identifying risk factors that may impair bonding is therefore essential for understanding vulnerability to bonding difficulties and for informing prevention and early intervention.

Although maternal bonding is a continuous process [7,21,22], it is often described as unfolding across two related periods. During pregnancy, a mother typically develops internalized representations of her unborn child, accompanied by an affectionate tie to the fetus, often referred to as antenatal maternal bonding [5]. Postpartum maternal bonding is the mother’s emotional tie to her child after birth, indicated by pleasure in interaction, developing competence in meeting infant needs, and acceptance of maternal role demands [6], and expressed through maternal sensitivity and emotional availability [23]. Condon (1993) proposes that antenatal bonding provides the essential structure for the subsequent development of postpartum bonding [5]. Supporting this theoretical framework, systematic reviews and prospective studies demonstrate that bonding strength across all trimesters predicts bonding quality during the postpartum period [21,24,25]. The postpartum period, however, introduces additional demands, including the day-to-day care of the infant and the mother’s physical recovery from childbirth [26,27], which may shape bonding in ways distinct from those during pregnancy. Therefore, antenatal and postpartum bonding cannot be assumed to be equivalent, making it important to examine the factors that predict bonding in each period separately.

Prior research most consistently links impaired bonding to perinatal depressive and anxiety symptoms [28,29,30], underscoring the relevance of affective distress to maternal bonding. Lower perceived social support has also been associated with poorer maternal bonding [31,32], suggesting that the broader interpersonal context may serve as a background factor. Much of this evidence, however, comes from cross-sectional studies or longitudinal studies that begin during pregnancy [29], making it difficult to capture factors that may predate conception. Indeed, risk factors relevant to bonding may originate well before pregnancy. Drawing on 20-year intergenerational cohort data, Olsson et al. (2021) found that depression and anxiety symptoms in adolescence predicted greater maternal–infant bonding difficulties years later [33]. Such long-term associations indicate that vulnerability to bonding difficulties is not confined to the perinatal period but may be rooted in characteristics already present long before conception [16,33]. Moreover, depressive and anxiety symptoms, although established correlates of bonding, may also co-occur with or stem from more stable dispositional vulnerabilities [34,35]. Identifying such vulnerabilities, rather than only their symptomatic expressions, may therefore be crucial for understanding bonding difficulties.

Among these dispositional factors, a highly relevant pre-pregnancy cognitive vulnerability is learned helplessness (LH). LH is characterized by generalized expectations that one’s actions cannot effectively influence desired outcomes, often developing after repeated exposure to uncontrollable experiences [36,37]. LH operates as a trait-like system of expectancies [38] and has been described as a relatively stable feature over the life course [39,40]. Contemporary perspectives further suggest that passivity in the face of uncontrollable adversity may reflect an automatic default response, whereas what is learned is controllability—the expectation that one’s actions can produce desired outcomes [41,42,43]. Consistent with this view, LH has been operationalized as a multifaceted, trait-like construct encompassing perceived lack of control, diminished self-efficacy, negative comparative self-evaluation, and the generalization of past experiences to future expectations, with these perceptions not tied to a specific time frame or situation [39]. A core consequence of LH is motivational withdrawal. When facing new demands or challenges, individuals who anticipate that effort will be ineffective may show reduced initiation of action and withdraw from active engagement, even in circumstances in which their behavior could influence outcomes [36,37].

LH may be particularly relevant to maternal bonding because the perinatal period requires sustained emotional and behavioral engagement under conditions that are often uncertain and only partially controllable [26,44,45,46]. During pregnancy, bonding involves forming representations of the fetus, oneself as a mother, and the anticipated mother–child relationship [5,47]. After birth, bonding is embedded in the daily demands of caregiving, including responding to infant cues, even when maternal efforts do not always produce the desired response [48,49]. For women with elevated LH, these contexts may activate generalized expectations that their actions have limited influence, reducing motivation to initiate or sustain engagement [36,37]. Because maternal bonding depends on active emotional investment, involvement, and pleasure in proximity to the fetus or infant [6,50], helplessness-related withdrawal may be especially relevant to lower bonding during both pregnancy and the postpartum period. At the same time, LH is closely associated with symptoms of depression and anxiety [39,42,51,52], which are among the most consistent correlates of impaired bonding [22,29,31].

In perinatal research, LH has received limited empirical attention. Although LH theory has informed conceptualizations of postpartum depression [53,54], and situational appraisals of helplessness have been linked to antenatal distress [55], LH has rarely been investigated as a stable preconception vulnerability in maternal bonding research. To our knowledge, no study has assessed LH before pregnancy or evaluated its prospective association with maternal bonding across pregnancy and the postpartum period. To address this gap, the present prospective study examined preconception LH as a cognitive vulnerability for lower maternal bonding across pregnancy and the postpartum period. Importantly, the study focused on a euthymic, non-clinical sample, offering an opportunity to examine whether LH, conceptualized as a relatively stable cognitive vulnerability, is relevant to maternal bonding in the absence of significant current psychopathology. We first examined the temporal stability of LH from preconception to postpartum and the continuity of bonding from antenatal to postpartum periods, as these analyses provided the conceptual basis for testing LH as a stable vulnerability and antenatal bonding as a mediator. We then tested two hypotheses. First, we hypothesized that higher preconception LH would predict lower antenatal and postpartum bonding beyond depression and anxiety symptoms assessed during pregnancy and postpartum, respectively. Second, we hypothesized that the association between preconception LH and postpartum bonding would be partially mediated by lower antenatal bonding. Prenatal depressive and anxiety symptoms were examined as additional parallel mediators, given their established associations with both LH and maternal bonding.

## 2. Materials and Methods

### 2.1. Study Design, Participants, and Recruitment

Data for the present study were drawn from a larger prospective study examining neural and psychological mechanisms underlying perinatal mental health among community-dwelling Israeli women. The study followed women from preconception through the early postpartum period. Recruitment occurred from November 2020 to October 2024. The study was approved by the Institutional Helsinki Committee of Hadassah Medical Center (Ein Kerem), Jerusalem, Israel (Protocol No. 0301-20-HMO; approval date: 18 August 2020). All participants provided written informed consent before participating. At recruitment, eligible participants were healthy, non-pregnant, nulliparous women aged 18–38 years who planned to conceive naturally, did not require fertility treatment, and did not take regular medications other than oral contraceptives or prenatal vitamins. Women were excluded if they had a prior pregnancy, significant medical or obstetric conditions, or did not meet psychiatric eligibility criteria. Psychiatric exclusion criteria included current major depression or major depression within the past two years, bipolar disorder, psychotic disorder, active suicidality, and substance use disorder.

A total of 420 women completed an initial telephone screening, of whom 195 were excluded based on medical, obstetric, fertility-related, medication-related, or prior-pregnancy criteria. The remaining 225 women underwent a semi-structured psychiatric interview to confirm psychiatric eligibility and euthymic status, and 17 were excluded based on psychiatric criteria. Thus, 208 euthymic, nulliparous women were enrolled at the preconception baseline. Of these, 34 withdrew before providing study data, and 23 were excluded from the present analyses because they lacked preconception LH data or did not provide a maternal bonding assessment. The final analytic sample comprised 151 women who completed the preconception LH assessment and provided at least one antenatal or postpartum bonding assessment. Participants who later required fertility treatment remained eligible for follow-up because eligibility was determined at enrollment. Detailed participant flow is presented in Supplementary Figure S1.

### 2.2. Procedure and Assessment Timeline

After enrollment, participants completed study questionnaires at five assessment points: preconception baseline (T0), each trimester of pregnancy (T1–T3), and the postpartum assessment (T4). At T0, participants completed measures of sociodemographic characteristics, LH, depressive symptoms, anxiety symptoms, and perceived social support. After pregnancy was confirmed, participants were assessed once per trimester. At T1, T2, and T3, they completed measures of antenatal bonding and depressive and anxiety symptoms. At T4, participants completed measures of LH, postpartum bonding, depressive symptoms, anxiety symptoms, and obstetric and neonatal outcomes. The postpartum assessment was scheduled for approximately 4–6 weeks after childbirth, though completion times varied across participants (*M* = 8.8 weeks, *SD* = 4.6; median = 7.7; range = 0.7–32.3 weeks). Throughout the manuscript, baseline refers to the preconception assessment; prenatal depressive and anxiety symptoms refer to symptoms assessed during pregnancy; and postpartum bonding refers to bonding assessed after birth.

### 2.3. Measures

#### 2.3.1. Psychiatric Screening

Psychiatric eligibility was assessed using the Hebrew version of the Diagnostic Interview for Anxiety, Mood, and Obsessive-Compulsive and Related Neuropsychiatric Disorders (DIAMOND; [56]), a semi-structured clinical interview that assesses psychiatric disorders according to the Diagnostic and Statistical Manual of Mental Disorders, Fifth Edition (DSM-5) [57], including mood, anxiety, obsessive-compulsive, trauma-related, psychotic, and substance-related disorders. The DIAMOND has demonstrated adequate interrater and test–retest reliability in its original validation study [56]. In the current study, trained clinicians administered the interview at screening to determine psychiatric eligibility and confirm euthymic status before enrollment.

#### 2.3.2. Sociodemographic, Obstetric, and Neonatal Information

Sociodemographic, obstetric, and neonatal information was collected via questionnaires at preconception baseline (T0) and postpartum (T4). At T0, participants reported sociodemographic characteristics, including age, years of education, relationship status, and household income. At T4, participants reported obstetric and neonatal outcomes, including conception modality, pregnancy course, gestational age at birth, mode of delivery, newborn sex, birthweight, neonatal intensive care unit (NICU) admission, and maternal postpartum hospitalization. These variables were used to characterize the analytic sample, and selected obstetric and neonatal variables were evaluated in sensitivity analyses to determine whether they warranted inclusion as covariates.

#### 2.3.3. Learned Helplessness

LH was assessed using the Learned Helplessness Questionnaire (LHQ; [39]), a 20-item Hebrew self-report measure that assesses psychological aspects of LH, including perceptions of control, self-efficacy beliefs, comparisons of personal well-being with that of others, and the generalization of past experiences to future expectations. Items are rated on a 4-point scale from 1 (very false) to 4 (very true), with total scores ranging from 20 to 80. Higher scores indicate higher LH levels. The LHQ has demonstrated adequate concurrent validity with the Learned Helplessness Scale (*r* = 0.70; [58]) and the Beck Hopelessness Scale (*r* = 0.50; [59]), as well as strong internal consistency across prior samples (*α* = 0.87–0.90; [39]). In the current sample, internal consistency was excellent across assessments, with Cronbach’s α ranging from 0.91 to 0.92. The LHQ was administered at the preconception baseline assessment (T0) and reassessed at the postpartum assessment (T4). Preconception LH assessed at T0 served as the primary predictor in the main analyses, whereas postpartum LH assessed at T4 was used in background analyses examining temporal stability.

#### 2.3.4. Maternal Bonding

Antenatal bonding was assessed at each pregnancy assessment (T1–T3) using the Maternal Antenatal Attachment Scale (MAAS; [5]). The MAAS is a 19-item self-report measure that assesses maternal emotional bonding with the fetus, including the quality and intensity of maternal–fetal bonding. Items are rated on a 5-point scale, with total scores ranging from 19 to 95. Higher scores indicate stronger antenatal bonding. In the current sample, the MAAS showed good internal consistency across pregnancy, with Cronbach’s *α* ranging from 0.81 to 0.82.

Postpartum bonding was assessed during the postpartum assessment (T4) using the Maternal Postnatal Attachment Scale (MPAS; [6]). The MPAS is a 19-item self-report measure that assesses maternal emotional bonding with the infant after birth, including pleasure in proximity, acceptance, tolerance, and competence. Items are rated on a 5-point scale, and total scores range from 19 to 95. Higher scores indicate stronger postpartum bonding. In the current sample, internal consistency was good, with Cronbach’s α = 0.83.

Although the original scales use the term “attachment,” both measures were used in the present study to index maternal bonding, defined as the mother’s affective tie to the fetus during pregnancy and to the infant after birth. MAAS total scores were calculated separately for each pregnancy assessment (T1–T3). When an aggregate index of antenatal bonding was required, a mean MAAS total score across T1–T3 was computed.

#### 2.3.5. Depressive and Anxiety Symptoms

Depressive symptoms were assessed using the Patient Health Questionnaire-9 (PHQ-9; [60]), a 9-item self-report measure of depression severity. Items are rated on a 4-point scale from 0 (not at all) to 3 (nearly every day), yielding total scores ranging from 0 to 27. Higher scores indicate greater depressive symptom severity. The Hebrew version of the PHQ-9 has been validated in primary care settings [61]. In the current sample, internal consistency was acceptable to good across assessment points, with Cronbach’s α ranging from 0.73 to 0.81.

Anxiety symptoms were assessed using the Generalized Anxiety Disorder-7 (GAD-7; [62]), a 7-item self-report measure of anxiety symptom severity. Items are rated on a 4-point scale from 0 (not at all) to 3 (nearly every day), with total scores ranging from 0 to 21. Higher scores indicate greater anxiety symptom severity. The official Hebrew version authorized by the Israeli Ministry of Health was used. In the current sample, internal consistency was good across assessment points, with Cronbach’s α ranging from 0.79 to 0.87. The PHQ-9 and GAD-7 were administered at all five assessments: preconception baseline (T0), each pregnancy assessment (T1–T3), and the postpartum assessment (T4).

#### 2.3.6. Perceived Social Support

Baseline perceived social support was assessed at the preconception baseline (T0) using a translated Hebrew version of the Social Support and Social Undermining Scale, originally developed by Abbey et al. (1985) [63] and later adapted by Howe et al. (2004) [64]. Participants identified the person they felt closest to and interacted with at least once a week, and rated that person’s behaviors toward them on a 5-point Likert scale from 1 (not at all) to 5 (a great deal). The measure includes items assessing perceived support and social undermining. In the current study, one undermining item was excluded due to a negative item–total correlation. The remaining undermining items were reverse-coded and averaged with the support items to create a composite index, with higher scores indicating a more supportive and less undermining interpersonal environment. This composite served as a baseline interpersonal covariate in adjusted sensitivity analyses. Internal consistency for the composite was acceptable, *α* = 0.79.

### 2.4. Data Preparation and Missing Data

Scale scores were computed according to the scoring instructions for each measure, with higher scores indicating higher levels of the respective construct. Preconception LH assessed at T0 served as the primary predictor in the main analyses, whereas postpartum LH assessed at T4 was used only in background analyses of temporal stability. Postpartum bonding was represented by the MPAS total score at T4. Antenatal bonding was represented in three ways to meet the analytic aim: trimester-specific MAAS total scores served as repeated measures in the linear mixed-effects model; a mean MAAS score across available T1–T3 assessments was used in correlational and mediation analyses; and MAAS scores from T1, T2, and T3 were modeled as indicators of a latent antenatal bonding construct in the SEM analysis. Mean prenatal depressive and anxiety symptom scores were computed by averaging PHQ-9 and GAD-7 scores, respectively, across available pregnancy assessments.

For longitudinal analyses of antenatal bonding, prenatal depressive and anxiety symptoms were decomposed into within-person and between-person components. Within-person deviations were computed by subtracting each participant’s mean symptom score across pregnancy from her symptom score in each trimester. Between-person symptom levels were represented by each participant’s mean depressive or anxiety symptom score across T1–T3. This approach distinguished trimester-specific symptom fluctuations from stable between-person differences across pregnancy. Baseline covariates for the adjusted models included age at baseline, years of education, baseline depressive and anxiety symptoms, and baseline perceived social support. Obstetric and perinatal variables were used to characterize the sample descriptively and were examined in additional checks to determine whether they warranted inclusion as covariates. Time from childbirth to completion of the postpartum questionnaire was calculated in days and converted to weeks for descriptive reporting. Missing-data handling varied by analytic model. Linear mixed-effects models used all available repeated observations. Structural equation and mediation models were estimated using full-information maximum likelihood under the missing-at-random assumption. Hierarchical regression analyses used complete cases for the variables included in each model.

### 2.5. Statistical Analysis

Statistical analyses were conducted in jamovi (Version 2.7; The jamovi project, 2025), which runs within the R statistical environment (Version 4.5; R Core Team, 2025). All statistical tests were two-tailed, and the alpha level was set at 0.05. Descriptive statistics were computed for sociodemographic, obstetric, neonatal, and primary study variables. Potential attrition bias was evaluated by comparing participants in the analytic sample with excluded participants who provided baseline data, using Welch’s independent-samples *t*-tests. Preliminary analyses examined distributional properties, missing-data patterns, univariate outliers, and multicollinearity; these checks are summarized in the descriptive results. Bivariate associations among the main study variables were assessed using Pearson correlations and reported in the correlation table.

Before testing the main hypotheses, two background analyses were conducted. First, the temporal stability of LH from the preconception baseline (T0) to the postpartum assessment (T4) was assessed using Pearson correlations, and mean-level changes in LH, depressive symptoms, and anxiety symptoms were examined using paired-samples *t*-tests. Wilcoxon signed-rank tests served as nonparametric sensitivity checks when appropriate. Second, the association between antenatal and postpartum bonding was examined using structural equation modeling (SEM) in the SEMLj module [65], which interfaces with the lavaan engine [66]. Antenatal bonding was modeled as a latent construct indicated by MAAS total scores at T1, T2, and T3 and specified to predict postpartum bonding at T4. Model fit was evaluated using the χ^2^ test, comparative fit index (CFI), Tucker–Lewis index (TLI), root mean square error of approximation (RMSEA), and standardized root mean square residual (SRMR).

To test whether preconception LH predicted antenatal bonding across pregnancy beyond prenatal depressive and anxiety symptoms, a linear mixed-effects model was fit using the GAMLj module [67]. MAAS total scores at T1, T2, and T3 served as repeated outcome measures nested within participants. A random intercept for participants was specified, and fixed effects included time, preconception LH, within-person deviations in prenatal depressive and anxiety symptoms, and between-person mean levels of these symptoms. Time was modeled as a categorical fixed effect, with T1 as the reference category. Degrees of freedom were estimated using the Satterthwaite approximation, and confidence intervals for fixed effects were derived from 5000 bootstrap resamples.

To test whether preconception LH predicted postpartum bonding beyond concurrent affective symptoms, a hierarchical linear regression was conducted with postpartum bonding at T4 as the dependent variable. Postpartum depressive and anxiety symptoms were entered in Step 1, and preconception LH was added in Step 2 to assess its incremental contribution. Model fit was evaluated using *R*^2^, change in *R*^2^ (Δ*R*^2^), and F-tests for model comparison. Standardized and unstandardized regression coefficients were reported.

To examine indirect pathways linking preconception LH to postpartum bonding, a path-analytic parallel multiple mediation model was estimated using the jAMM module. Preconception LH was specified as the predictor, and postpartum bonding at T4 as the outcome. Three parallel mediators were entered simultaneously: mean antenatal bonding, mean prenatal depressive symptoms, and mean prenatal anxiety symptoms across T1–T3. The model tested the unique indirect effect of each mediator while controlling for the others. Indirect effects were evaluated using 95% bootstrap percentile confidence intervals based on 5000 resamples. Unstandardized coefficients, standard errors, completely standardized coefficients, and confidence intervals were reported.

Sensitivity and follow-up analyses were conducted to clarify the robustness and interpretation of the main findings. To assess robustness, the main antenatal bonding model, postpartum bonding regression, and mediation model were rerun with additional adjustment for age at baseline, years of education, baseline depressive and anxiety symptoms, and baseline perceived social support. Additional checks examined whether postpartum assessment timing and obstetric or perinatal variables were associated with bonding outcomes and therefore warranted inclusion as covariates. Follow-up mixed-effects models examined whether prenatal depressive and anxiety symptoms predicted antenatal bonding after excluding preconception LH. Symptoms were tested both simultaneously and separately. Detailed results are reported in the Supplementary Materials.

## 3. Results

### 3.1. Sample Characteristics

Sociodemographic and selected obstetric and neonatal characteristics of the analytic sample are presented in Table 1. The analytic sample comprised 151 nulliparous women recruited before conception. Participants were, on average, 25.18 years old (*SD* = 3.80, range = 19–38) and had completed 14.98 years of education (*SD* = 2.05, range = 12–20). Most participants were married (93.3%) or cohabiting (6.7%), and the majority reported below-average household income. Perinatal data were available for participants who had reached the relevant follow-up assessments by the time of analysis. Therefore, valid sample sizes varied across obstetric and neonatal variables. Among participants with available data, most pregnancies were uncomplicated (83.2%), mean gestational age at birth was 39.18 weeks (*SD* = 1.96, range = 25–42), most births were vaginal (70.8%), and 12 newborns (8.8%) were admitted to the neonatal intensive care unit (NICU).

### 3.2. Attrition Analysis

To assess potential attrition bias, participants in the analytic sample (*n* = 151) were compared with excluded participants who provided baseline data. Analyses used available baseline data, and the number of excluded participants varied by variable (*n* = 14–19). As shown in Supplementary Table S1, no significant group differences were observed for age, years of education, baseline depressive or anxiety symptoms, perceived social support, or preconception LH, with all *ps* ≥ 0.178. In addition, within the analytic sample, participants with available postpartum bonding data (*n* = 133) did not differ from those without such data (*n* = 18) in preconception LH, Welch’s *t* (23.65) = −0.45, *p* = 0.654, or in age, education, baseline depressive or anxiety symptoms, or perceived social support (all *ps* ≥ 0.278).

### 3.3. Descriptive Statistics of Main Study Variables and Preliminary Analyses

Descriptive statistics for the main study variables across assessment points are presented in Table 2. Mean depressive and anxiety symptom scores were generally low throughout the perinatal period. Antenatal bonding scores increased across pregnancy, whereas mean LH scores were similar at the preconception and postpartum assessments.

Continuous variables showed acceptable distributions, with skewness and kurtosis within conventional thresholds for univariate normality (|*skewness*| < 2, |*kurtosis*| < 7). Postpartum depressive symptoms showed elevated *kurtosis* (5.26), but this value remained within the acceptable range. Five cases had extreme values (|*z*| > 3.29) on one or more affective symptom measures; because these values were within the possible scale ranges and did not reflect data entry errors, they were retained. Multicollinearity was not a concern, with all variance inflation factor (VIF) values in the most complex model ≤2.52. Missing data were minimal at baseline (<2% for all preconception variables). Variation in sample size across follow-up assessments reflected the cohort’s ongoing nature, incomplete follow-up, and limited formal attrition; among participants in the analytic sample, three (2.0%) formally withdrew during follow-up.

### 3.4. Bivariate Correlations

Bivariate correlations among the main study variables are presented in Table 3. Preconception LH was positively associated with depressive and anxiety symptoms across assessment periods and negatively associated with mean antenatal bonding, *r* = −0.27, *p* < 0.001, and with postpartum bonding, *r* = −0.47, *p* < 0.001. Mean antenatal bonding was positively associated with postpartum bonding, *r* = 0.49, *p* < 0.001. Perceived social support was negatively associated with preconception LH and positively associated with both bonding outcomes. Overall, the correlation pattern supported further testing of whether preconception LH predicted antenatal and postpartum bonding beyond affective symptoms and whether antenatal bonding served as a pathway linking preconception LH to postpartum bonding.

### 3.5. Background Analyses

#### 3.5.1. Temporal Stability and Mean-Level Change in LH and Affective Symptoms

As a background analysis, we examined the stability of LH from preconception (T0) to the postpartum assessment (T4). Depressive and anxiety symptoms were assessed over the same interval for comparison. LH showed high rank-order stability from T0 to T4, *r* = 0.71, 95% CI [0.62, 0.79], *p* < 0.001. Depressive symptoms, *r* = 0.39, 95% CI [0.24, 0.53], *p* < 0.001, and anxiety symptoms, *r* = 0.50, 95% CI [0.36, 0.61], *p* < 0.001, also showed significant rank-order stability. Mean-level changes from T0 to T4 were examined using paired-samples *t*-tests among participants with complete data at both assessments. LH levels did not change significantly from preconception (*M* = 37.15, *SD* = 8.65) to postpartum (*M* = 37.46, *SD* = 8.61), *t*(133) = −0.55, *p* = 0.581, 95% CI [−1.43, 0.81], *d* = −0.05. In contrast, depressive symptoms increased significantly from preconception (*M* = 4.13, *SD* = 3.27) to postpartum (*M* = 4.90, *SD* = 4.16), *t*(134) = −2.17, *p* = 0.032, 95% CI [−1.49, −0.07], *d* = −0.19. Anxiety symptoms also increased significantly from preconception (*M* = 2.95, *SD* = 2.75) to postpartum (*M* = 3.63, *SD* = 3.70), *t*(135) = −2.36, *p* = 0.020, 95% CI [−1.24, −0.11], *d* = −0.20. Because the normality assumption for paired differences was violated for depressive and anxiety symptoms, Wilcoxon signed-rank tests were conducted as nonparametric sensitivity checks. These tests yielded the same pattern: depressive symptoms, *W* = 2381, *p* = 0.023, and anxiety symptoms, *W* = 2233, *p* = 0.029, but no significant change in LH, *W* = 3767, *p* = 0.569.

#### 3.5.2. Association Between Antenatal and Postpartum Bonding

The SEM model examining antenatal bonding as a latent construct, indicated by MAAS total scores at T1–T3, showed excellent fit to the data, χ^2^(2) = 0.70, *p* = 0.704, CFI = 1.00, TLI = 1.02, RMSEA = 0.000, SRMR = 0.010. Standardized factor loadings were high, ranging from λ = 0.80 to *λ* = 0.93, supporting a common antenatal bonding construct across pregnancy. Latent antenatal bonding significantly predicted postpartum bonding, β = 0.523, 95% CI [0.387, 0.659], *z* = 5.91, *p* < 0.001, and explained 27.3% of the variance in postpartum bonding. This association supported examining antenatal bonding as a mediator in the subsequent path model.

### 3.6. Main Hypothesis Tests

#### 3.6.1. Preconception LH and Antenatal Bonding Across Pregnancy

The linear mixed-effects model predicting antenatal bonding across pregnancy is presented in Table 4. Antenatal bonding increased significantly across pregnancy, *F*(2, 258) = 121.79, *p* < 0.001. As hypothesized, higher preconception LH predicted lower antenatal bonding across pregnancy, *b* = −0.20, *SE* = 0.06, 95% CI [−0.33, −0.08], *t*(145) = −3.16, *p* = 0.002. Neither within-person fluctuations nor between-person mean levels of prenatal depressive or anxiety symptoms were significant predictors in the full model, all *ps* > 0.20. Follow-up models examining the nonsignificant symptom effects are reported in the sensitivity and follow-up analyses section.

#### 3.6.2. Preconception LH and Postpartum Bonding

The hierarchical linear regression predicting postpartum bonding is presented in Table 5. The Step 1 model, which included postpartum depressive and anxiety symptoms, was significant, *R*^2^ = 0.327, *F*(2, 129) = 31.3, *p* < 0.001; both symptoms predicted lower postpartum bonding. Adding preconception LH in Step 2 significantly improved model fit, Δ*R*^2^ = 0.066, *F*(1, 128) = 14.0, *p* < 0.001. The final model explained 39.3% of the variance in postpartum bonding, *R*^2^ = 0.393, *F*(3, 128) = 27.6, *p* < 0.001. In the final model, higher preconception LH significantly predicted lower postpartum bonding, *b* = −0.29, *SE* = 0.08, 95% CI [−0.44, −0.13], β = −0.281, *p* < 0.001. Postpartum anxiety symptoms also remained a significant negative predictor, β = −0.301, *p* = 0.004, whereas postpartum depressive symptoms were no longer statistically significant, β = −0.190, *p* = 0.067. Thus, preconception LH explained significant incremental variance in postpartum bonding beyond concurrent depressive and anxiety symptoms.

#### 3.6.3. Indirect Pathways Linking Preconception LH to Postpartum Bonding

**Figure 1:**
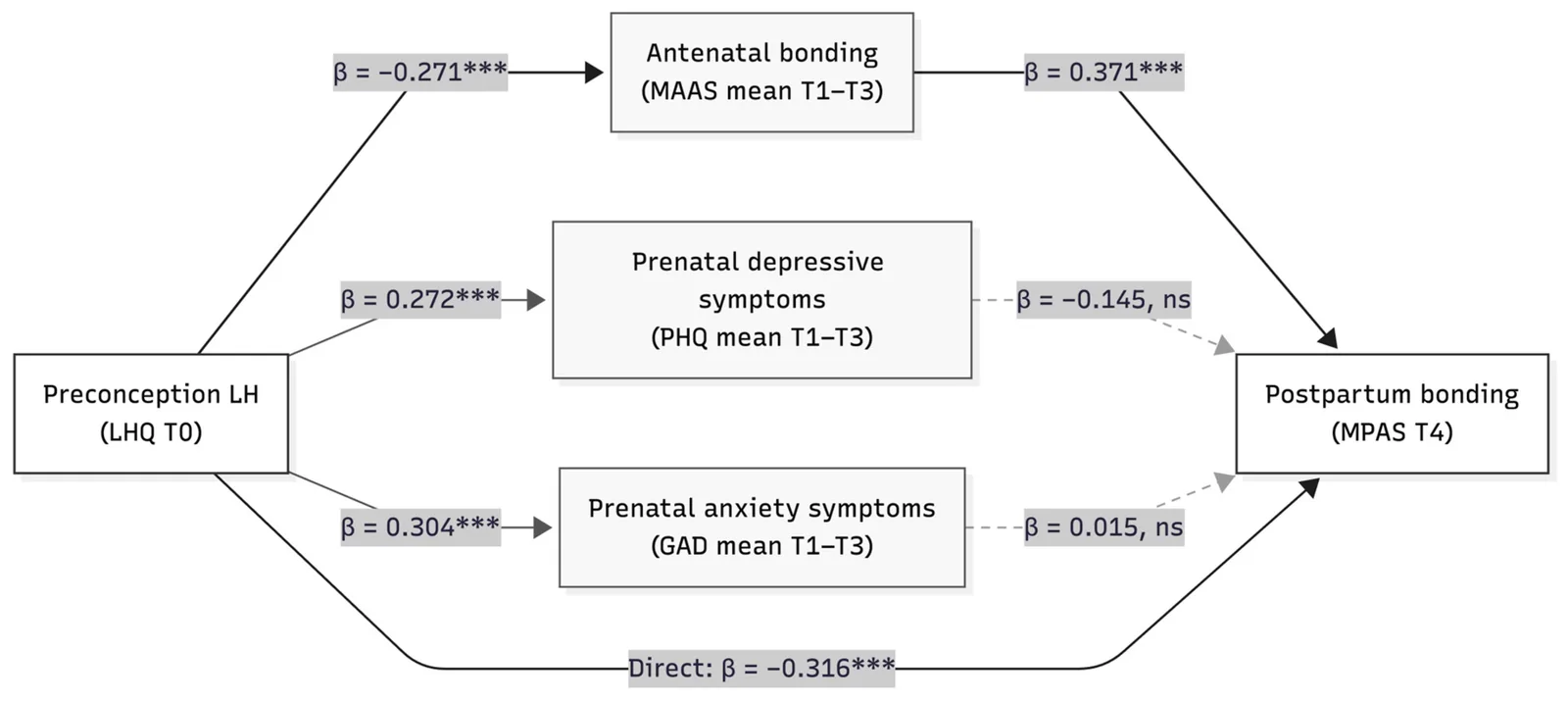
Parallel Mediation Model of the Association Between Preconception LH and Postpartum Bonding. Note. *N* = 151. Values are standardized path coefficients. Antenatal bonding, prenatal depressive symptoms, and prenatal anxiety symptoms were indexed as mean scores across T1–T3 assessments. Solid arrows indicate significant paths; dashed arrows indicate nonsignificant paths. LH = learned helplessness; MAAS = Maternal Antenatal Attachment Scale; MPAS = Maternal Postnatal Attachment Scale; T1–T3 = pregnancy assessments; T4 = postpartum assessment. *** *p* < 0.001; ns = not significant.

The parallel mediation model, shown in Figure 1, examined antenatal bonding, prenatal depressive symptoms, and prenatal anxiety symptoms as simultaneous mediators of the association between preconception LH and postpartum bonding; full estimates are reported in Supplementary Table S2. The total effect of preconception LH on postpartum bonding was significant, *b* = −0.475, *SE* = 0.078, 95% CI [−0.657, −0.314], β = −0.463, *p* < 0.001. The direct effect remained significant after accounting for the three mediators, *b* = −0.324, *SE* = 0.078, 95% CI [−0.507, −0.153], β = −0.316, *p* < 0.001, consistent with partial mediation. The indirect effect through antenatal bonding was significant, *b* = −0.103, *SE* = 0.037, 95% bootstrap CI [−0.188, −0.042], β = −0.101, *p* = 0.005. In contrast, the indirect effects through prenatal depressive symptoms, *b* = −0.040, *SE* = 0.030, 95% bootstrap CI [−0.122, 0.016], β = −0.039, *p* = 0.181, and prenatal anxiety symptoms, *b* = 0.005, *SE* = 0.031, 95% bootstrap CI [−0.053, 0.073], β = 0.005, *p* = 0.882, were not significant. Thus, the association between preconception LH and postpartum bonding was partially mediated by antenatal bonding but not by prenatal depressive or anxiety symptoms.

### 3.7. Sensitivity and Follow-Up Analyses

Detailed sensitivity and follow-up analyses are reported in Supplementary Text S1. In brief, LH-related findings remained robust after additional adjustment for age at baseline, years of education, baseline depressive and anxiety symptoms, and baseline perceived social support. Preconception LH remained a significant predictor of lower antenatal bonding, *b* = −0.199, *p* = 0.003, and lower postpartum bonding, *b* = −0.289, *p* < 0.001. The indirect effect through antenatal bonding also remained significant, *b* = −0.103, *p* = 0.008, whereas indirect effects through prenatal depressive and anxiety symptoms remained nonsignificant. Given the variability in postpartum assessment timing, we examined whether the number of days from childbirth to assessment was associated with postpartum bonding. No association was observed (Pearson *r*(131) = 0.01, *p* = 0.904; Spearman ρ = −0.01, *p* = 0.946), supporting the retention of assessments across the observed postpartum window. Obstetric and perinatal variables likewise did not warrant inclusion as additional covariates. Finally, follow-up models excluding preconception LH showed that prenatal depressive and anxiety symptoms were not robustly associated with antenatal bonding (Supplementary Table S3).

## 4. Discussion

The present longitudinal study examined preconception LH as a cognitive vulnerability to lower maternal bonding across pregnancy and the postpartum period. LH showed high temporal stability from preconception through the postpartum period, supporting its conceptualization as a relatively stable trait present before pregnancy. Consistent with our hypotheses, higher preconception LH was prospectively associated with lower antenatal and postpartum bonding, even after accounting for depressive and anxiety symptoms assessed during pregnancy and the postpartum period, respectively. The findings further showed that antenatal bonding was strongly correlated with postpartum bonding and partially mediated the association between preconception LH and postpartum bonding, whereas prenatal depressive and anxiety symptoms did not.

A central premise of this study was that LH represents a relatively stable cognitive vulnerability present before pregnancy. The current findings support this premise and, to our knowledge, are the first to demonstrate the temporal stability of LH from preconception through the postpartum period. Women who reported higher LH before pregnancy tended to report higher LH again postpartum, while the sample’s average LH level remained stable across this period. In contrast, depressive and anxiety symptoms increased significantly, consistent with the heightened affective vulnerability characteristic of the perinatal period [68,69]. This pattern helps distinguish LH from the affective symptoms with which it is commonly associated [34] and supports its conceptualization as a trait-like vulnerability rather than a state-dependent response. This interpretation aligns with evidence that helplessness-related perceptions of uncontrollability can remain stable over time [38,40,70] and parallels findings that early maladaptive schemas show long-term stability independent of symptom severity [71].

The finding that higher preconception LH was prospectively associated with lower antenatal and postpartum bonding may be understood in light of LH’s core features. LH involves generalized expectations that desired outcomes are not effectively influenced by one’s own actions and has been operationalized as including diminished self-efficacy and a perceived lack of control [37,39]. In the perinatal context, such expectations may undermine a woman’s sense of her ability to provide effective care, foster closeness, and influence the developing relationship with her fetus or infant. This may reduce emotional engagement and increase withdrawal from bonding-related thoughts, feelings, and interactions [36,37]. Because maternal bonding itself involves emotional investment, mental preoccupation, pleasure in proximity, and perceived parental competence [5,6], LH-related expectations may compromise several central components of the bonding process and thereby contribute to lower maternal bonding.

During pregnancy, LH-related expectations may be expressed primarily in the representational domain before the mother–infant relationship is enacted through direct caregiving. Before birth, the mother forms an emotional tie to a child she has not yet met while developing mental representations of the fetus, herself as a mother, and the future mother–child relationship [5,47]. These representations encompass expectations about her maternal role, agency, and capacity to influence the emerging relationship. Because LH involves expectations of limited control and diminished efficacy, it may shape these representations toward a view of the self as less capable, less influential, or less able to foster closeness within the emerging maternal relationship. This may contribute to less frequent or less positive mental engagement with the fetus, which is central to antenatal bonding [5]. This interpretation aligns with research linking maternal self-efficacy and perceived maternal capability to stronger maternal–fetal bonding [72,73,74]. Notably, preconception LH remained associated with lower antenatal bonding even when prenatal depressive and anxiety symptoms were included in the model, whereas prenatal affective symptoms did not show robust associations with antenatal bonding. This pattern suggests that LH may capture a vulnerability to lower antenatal bonding beyond concurrent prenatal affective symptom severity.

After birth, LH may manifest more behaviorally in the context of direct caregiving. The early postpartum period often involves substantial physical and emotional strain, including fatigue and the ongoing demands of infant care [26,49]. In this context, infant distress signals, such as prolonged crying or a difficult temperament, do not always respond to maternal soothing efforts [48,53]. For women with higher LH, repeated experiences in which caregiving efforts do not yield desired outcomes may reinforce the expectation that their actions have limited influence over outcomes and contribute to the motivational deficit characteristic of helplessness, including reduced initiation of effort and withdrawal from active engagement [36,37]. Reduced engagement, in turn, may limit opportunities for responsive, mutually rewarding interactions, which are central to strengthening the maternal–infant bond [29,50].

The mediation findings further suggest that the association between preconception LH and postpartum bonding is partly rooted in bonding processes evident during pregnancy. Antenatal bonding showed strong continuity with postpartum bonding, consistent with the view that the antenatal bond provides an important foundation for the maternal–infant bond that follows birth [5,22,24,25]. Lower antenatal bonding partially accounted for the association between preconception LH and postpartum bonding, whereas prenatal depressive and anxiety symptoms did not. This pattern suggests that among women with higher preconception LH, lower postpartum bonding may partly reflect a pattern already evident during pregnancy, rather than difficulties emerging only after birth. At the same time, preconception LH remained directly associated with postpartum bonding even after accounting for antenatal bonding, suggesting that additional postpartum processes may also be involved. These processes may include a mother’s perceived caregiving competence [73,75] and perceived capacity and resources to meet the physical and emotional demands of early motherhood [26,27]. These possibilities were not directly tested in the present study, but they may point to important directions for future research.

In the primary postpartum regression model, concurrent depressive and anxiety symptoms initially predicted lower postpartum bonding. After preconception LH was added to the model, the association with postpartum depressive symptoms was attenuated and no longer statistically significant, whereas postpartum anxiety symptoms retained a unique contribution. This pattern aligns with extensive evidence linking postpartum depression and anxiety to weaker maternal bonding [22,28,29] and suggests that LH may account for part of the bonding-relevant variance otherwise associated with depressive symptoms. The attenuation of the depressive association may reflect conceptual overlap between LH and depression [36,37]. By contrast, postpartum anxiety symptoms may operate through partially distinct processes. Rather than withdrawal, anxiety is characterized by heightened threat sensitivity and hyperarousal [76], which in caregiving may manifest as hypervigilance, over-involvement, or high-arousal parenting that is poorly attuned to the infant’s cues [77,78,79]. These symptom-specific interpretations should be treated cautiously, as the present study did not directly test these mechanisms. Overall, these findings position LH as a stable preconception vulnerability in models of maternal bonding, without diminishing the clinical importance of concurrent postpartum distress.Clinically, these findings suggest that assessing LH may complement standard screening for perinatal depression and anxiety by capturing expectations of low control and diminished efficacy that are not necessarily reflected in current symptom severity [35]. Because LH was assessed before conception and remained stable throughout the perinatal period, elevated LH may help identify women vulnerable to poorer maternal bonding, even when affective symptoms are mild or absent, underscoring its potential relevance in non-clinical populations. The findings also suggest potential intervention targets and indicate that women with elevated LH may benefit from approaches that directly address helplessness-related expectations by fostering learned controllability, the belief that one’s actions can produce desired outcomes [42,43]. Cognitive-behavioral approaches that challenge maladaptive beliefs and strengthen perceived control may therefore be relevant for women with elevated LH [35,80], particularly when combined with support for maternal emotional engagement with the fetus [3,29,74]. The potential significance of these findings may also extend beyond maternal well-being to offspring development. Prospective evidence indicates that stronger antenatal and early postnatal bonding is associated with more favorable infant temperament and social–emotional outcomes [3,13]. Longitudinal studies also link maternal bonding to broader aspects of early childhood development, including cognitive and motor development [7,81]. By identifying preconception LH as a vulnerability linked to lower bonding beginning in pregnancy, the present findings raise the possibility that its relevance may extend beyond mothers’ bonding experiences to later child developmental outcomes. Because offspring outcomes were not assessed in the present study, this possibility remains to be tested directly in longitudinal studies examining whether preconception LH and subsequent bonding trajectories are associated with later child development.

Several limitations should be considered. First, the primary constructs were assessed using self-report questionnaires, which may reflect participants’ perceptions [78,82]. In addition, the proposed mechanisms linking LH to lower bonding, including perceived maternal effectiveness, emotional withdrawal, and reduced caregiving engagement, were not directly assessed. Future studies could complement self-report measures with interview-based assessments of maternal representations during pregnancy and with observational assessments of mother–infant interaction after birth [82,83]. Second, although the sample size was substantial for a prospective study beginning before conception, it was still modest for testing complex pathways and smaller indirect effects; replication in larger samples is therefore needed. Third, the sample consisted of screened, euthymic, nulliparous women, most of whom were highly educated and in stable relationships. Although most reported below-average household income, this sample profile may limit generalizability to populations experiencing greater socioeconomic and psychosocial adversity. Socioeconomic disadvantage and limited social or partner support are established risk factors for perinatal mental health difficulties [84,85,86,87] and have also been linked to poorer maternal–infant bonding [85,87]. The associations observed here may therefore differ among women experiencing greater financial strain, social adversity, or limited support and should be examined in more socioeconomically and clinically diverse samples. Relatedly, depressive and anxiety symptoms were generally low, which may have restricted variability in symptom–bonding associations. Finally, although the prospective preconception design strengthens temporal ordering, causal conclusions cannot be drawn.

## 5. Conclusions

This prospective study identifies preconception LH as a relatively stable cognitive vulnerability relevant to maternal bonding throughout pregnancy and the postpartum period. Beyond its association with concurrent affective distress, LH appears to reflect a preexisting pattern of low perceived control and diminished efficacy, which may shape how women enter the maternal role and engage in the emerging maternal bond. The finding that antenatal bonding partially mediated the association between preconception LH and postpartum bonding suggests that LH’s relevance begins before birth, as the maternal bond is first represented and emotionally elaborated during pregnancy. Therefore, the study extends models of maternal bonding by highlighting preconception LH as a cognitive vulnerability in the development of the maternal bond. Clinically, these findings support complementing symptom-based screening with attention to helplessness-related expectations before or early in pregnancy, and point to perceived control, maternal efficacy, and antenatal bonding as promising targets for future intervention research.

## Figures and Tables

**Table 1:** Sociodemographic, Obstetric, and Neonatal Characteristics of the Analytic Sample (*N* = 151).

Variable	*n*	M (SD)	Range	*n* (%)
**Sociodemographic characteristics**				
Age at baseline	151	25.18 (3.80)	19–38	
Years of education	148	14.98 (2.05)	12–20	
**Relationship status**	149			
Married				139 (93.3)
Cohabiting				10 (6.7)
**Household income**	149			
Below average				93 (62.4)
Average				21 (14.1)
Above average				35 (23.4)
**Obstetric and neonatal outcomes**				
**Pregnancy course**	137			
Without complications				114 (83.2)
With complications				23 (16.8)
Gestational age at birth, weeks	137	39.18 (1.96)	25–42	
**Mode of delivery**	137			
Vaginal delivery				97 (70.8)
Assisted vaginal delivery				3 (2.2)
Planned cesarean section				16 (11.7)
Emergency cesarean section				21 (15.3)
**Prolonged maternal postpartum hospitalization**	136			
No				103 (75.7)
Yes				33 (24.3)
**Newborn sex**	137			
Male				76 (55.5)
Female				61 (44.5)
Birthweight, g	137	3178.71 (547.37)	780–4350	
**NICU admission**	137			
No				125 (91.2)
Yes				12 (8.8)

Note. The *n* column indicates the number of participants with available data for each variable. Percentages are based on available data for each variable. Household income was reported relative to the national average. Pregnancy course was categorized as with or without complications based on the presence of any reported pregnancy complication. Maternal postpartum hospitalization refers to prolonged hospitalization after childbirth. Sociodemographic data were collected at T0, and obstetric and neonatal outcomes were collected at T4. NICU = neonatal intensive care unit; T0 = preconception baseline; T4 = postpartum assessment.

**Table 2:** Descriptive Statistics for Main Study Variables Across Assessment Points (*N* = 151).

Variable	*n*	M (SD)	Range
**Baseline measures (T0)**			
Learned helplessness	151	37.10 (8.51)	22–66
Depressive symptoms	150	4.09 (3.14)	0–17
Anxiety symptoms	150	2.93 (2.67)	0–10
Perceived social support	151	81.40 (5.40)	60–90
**Pregnancy measures (T1–T3)**			
**Antenatal bonding**			
Trimester 1	143	71.16 (7.96)	41–89
Trimester 2	135	75.74 (7.10)	55–91
Trimester 3	126	79.31 (6.56)	62–93
Mean T1–T3	150	75.09 (6.63)	57.7–88.3
**Depressive symptoms**			
Trimester 1	143	7.49 (4.38)	0–22
Trimester 2	135	5.79 (3.85)	0–19
Trimester 3	127	5.71 (3.79)	0–21
Mean T1–T3	150	6.39 (3.44)	0–17
**Anxiety symptoms**			
Trimester 1	143	3.75 (3.30)	0–15
Trimester 2	135	3.79 (3.24)	0–14
Trimester 3	126	3.43 (3.29)	0–14
Mean T1–T3	150	3.57 (2.76)	0–13.7
**Postpartum measures (T4)**			
Postpartum bonding	133	79.00 (8.76)	52.1–95.0
Learned helplessness	134	37.46 (8.61)	20–58
Depressive symptoms	136	4.91 (4.14)	0–25
Anxiety symptoms	137	3.66 (3.71)	0–19

Note. The *n* column indicates the number of participants with available data for each variable. Mean T1–T3 scores were calculated across all available pregnancy assessments. T0 = preconception baseline; T1–T3 = pregnancy assessments; T4 = postpartum assessment.

**Table 3:** Bivariate Correlations Among Main Study Variables (*N* = 151).

Variable	1	2	3	4	5	6	7	8	9	10
1. Learned helplessness (T0)	—									
2. Depressive symptoms (T0)	0.349 ***	—								
3. Anxiety symptoms (T0)	0.389 ***	0.712 ***	—							
4. Perceived social support (T0)	−0.313 ***	−0.177 *	−0.256 **	—						
5. Prenatal depressive symptoms (T1–T3)	0.273 ***	0.379 ***	0.341 ***	−0.158	—					
6. Prenatal anxiety symptoms (T1–T3)	0.305 ***	0.458 ***	0.523 ***	−0.242 **	0.677 ***	—				
7. Antenatal bonding (T1–T3)	−0.272 ***	0.023	0.020	0.348 ***	−0.165 *	−0.095	—			
8. Depressive symptoms (T4)	0.380 ***	0.392 ***	0.389 ***	−0.139	0.503 ***	0.505 ***	−0.331 ***	—		
9. Anxiety symptoms (T4)	0.364 ***	0.428 ***	0.495 ***	−0.253 **	0.460 ***	0.534 ***	−0.309 ***	0.731 ***	—	
10. Postpartum bonding (T4)	−0.470 ***	−0.238 **	−0.233 **	0.323 ***	−0.313 ***	−0.247 **	0.487 ***	−0.518 ***	−0.541 ***	—

Note. Pearson correlations are presented. Prenatal depressive and anxiety symptoms and antenatal bonding were indexed as mean scores across available T1–T3 assessments. Pairwise Ns ranged from 132 to 151 due to missing data. T0 = preconception baseline; T1–T3 = pregnancy assessments; T4 = postpartum assessment. * *p* < 0.05. ** *p* < 0.01. *** *p* < 0.001.

**Table 4:** Linear Mixed-Effects Model Predicting Antenatal Bonding Across Pregnancy (*N* = 150).

Predictor	b	SE	95% CI	t	df	*p*
**Fixed Effects**						
(Intercept)	71.27	0.59	[70.11, 72.41]	121.27	230	<0.001
Time (T2 vs. T1)	4.42	0.50	[3.46, 5.38]	8.92	257	<0.001
Time (T3 vs. T1)	7.89	0.51	[6.91, 8.88]	15.58	259	<0.001
Preconception LH	−0.20	0.06	[−0.33, −0.08]	−3.16	145	0.002
Prenatal depressive symptoms (WP) ^a^	−0.10	0.10	[−0.29, 0.09]	−0.99	252	0.322
Prenatal anxiety symptoms (WP) ^a^	−0.14	0.12	[−0.39, 0.10]	−1.15	251	0.251
Prenatal depressive symptoms (Mean) ^b^	−0.26	0.21	[−0.68, 0.15]	−1.26	154	0.208
Prenatal anxiety symptoms (Mean) ^b^	0.18	0.26	[−0.32, 0.69]	0.70	150	0.486
**Random Effects**	Variance	*SD*	ICC			
Participant (Intercept)	34.35	5.86	0.70			
Residual	14.80	3.84				

Note. The model included 404 observations from 150 participants. Time was modeled as a categorical fixed effect, with T1 as the reference category. WP = within-person; ^a^ Within-person deviations from each participant’s mean symptom level across pregnancy. ^b^ Between-person mean symptom level across T1–T3. *b* = unstandardized coefficient; *SE* = standard error; CI = 95% bootstrap confidence interval based on 5000 resamples; *df* = degrees of freedom (Satterthwaite approximation); LH = learned helplessness. Marginal *R*^2^ = 0.233, Conditional *R*^2^ = 0.769.

**Table 5:** Hierarchical Regression Predicting Postpartum Bonding (*N* = 132).

Predictor	b	SE	95% CI	β	t	*p*
**Step 1**						
Intercept	84.78	0.98	[82.83, 86.72]	—	86.20	<0.001
Postpartum depressive symptoms	−0.54	0.22	[−0.98, −0.10]	−0.257	−2.42	0.017
Postpartum anxiety symptoms	−0.83	0.25	[−1.32, −0.34]	−0.356	−3.35	0.001
**Step 2**						
Intercept	94.23	2.70	[88.90, 99.57]	—	34.95	<0.001
Postpartum depressive symptoms	−0.40	0.22	[−0.83, 0.03]	−0.190	−1.85	0.067
Postpartum anxiety symptoms	−0.70	0.24	[−1.17, −0.23]	−0.301	−2.95	0.004
Preconception LH	−0.29	0.08	[−0.44, −0.13]	−0.281	−3.74	<0.001

Note. Postpartum bonding was assessed using MPAS total scores at T4. *b* = unstandardized coefficient; *SE* = standard error; CI = confidence interval; β = standardized coefficient; LH = learned helplessness. Step 1: *R*^2^ = 0.327, *F*(2, 129) = 31.3, *p* < 0.001. Step 2: *R*^2^ = 0.393, *F*(3, 128) = 27.6, *p* < 0.001. Change from Step 1 to Step 2: Δ*R*^2^ = 0.066, *F*(1, 128) = 14.0, *p* < 0.001.

## Data Availability

The data presented in this study are available upon reasonable request from the corresponding author. The data are not publicly available due to privacy restrictions.

## Supplementary Materials

The following supporting information can be downloaded at: https://www.mdpi.com/article/10.3390/jcm15166391/s1. Figure S1: Participant flow through the study; Table S1: Baseline Comparisons Between Included and Excluded Participants; Text S1: Sensitivity and Follow-Up Analyses; Table S2: Parallel Mediation Model of the Association Between Preconception LH and Postpartum Bonding (*N* = 151); Table S3: Follow-Up Mixed-Effects Models Examining Prenatal Affective Symptoms as Predictors of Antenatal Bonding After Excluding Preconception LH.
