# Supplementary material for: Preconception Learned Helplessness as a Cognitive Vulnerability to Lower Maternal Bonding Across the Perinatal Period
Source: J Clin Med. 2026 Aug 18;15(16):6391. doi: 10.3390/jcm15166391 (PMC13513843; doi:10.3390/jcm15166391)
Supplement: Supplementary file 1 [file jcm-15-06391-s001.zip › jcm-4473429-supplementary.pdf]

Supplementary Materials

Supplementary Figure S1

Participant Flow through the Study

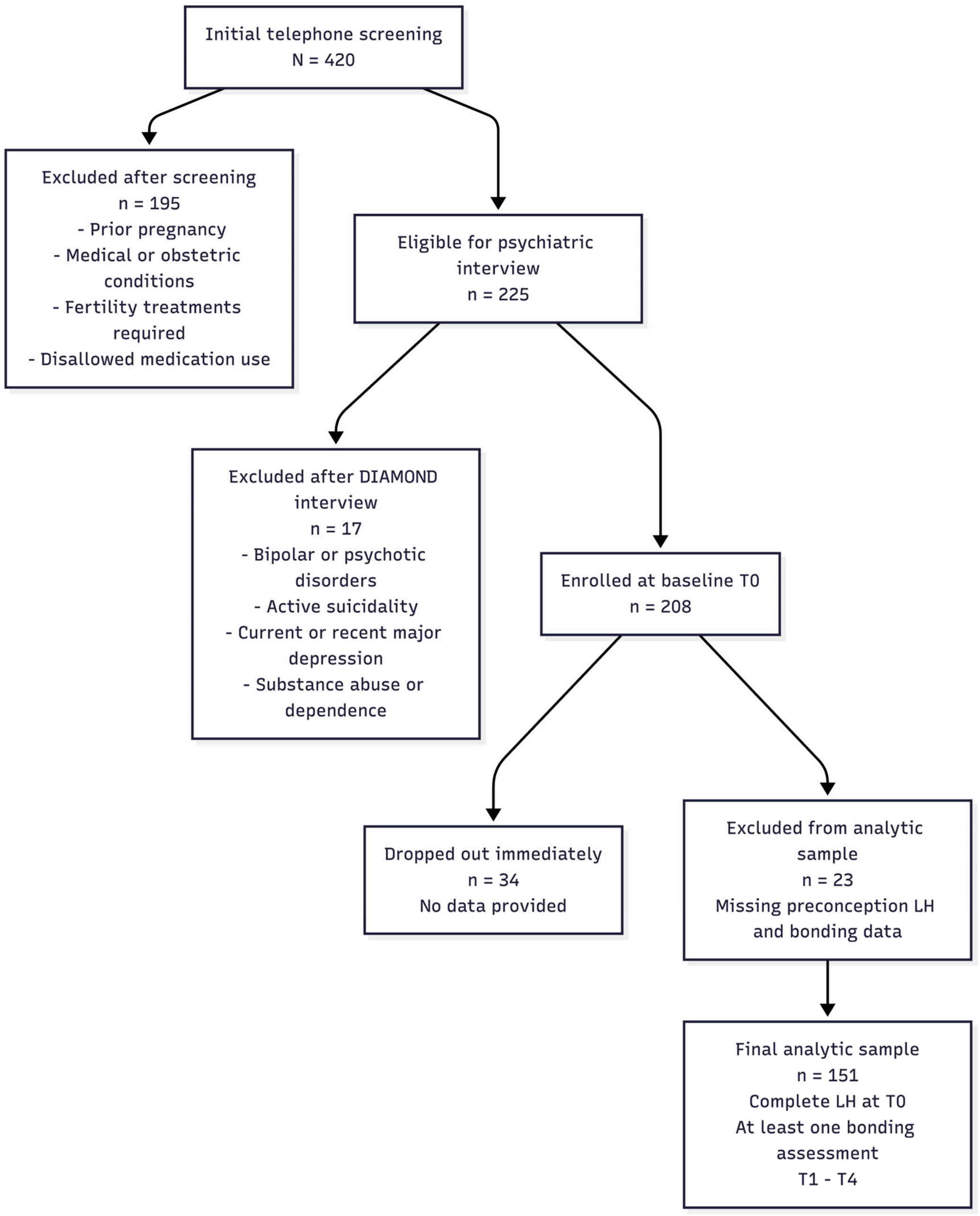

Note. LH = learned helplessness; T0 = preconception baseline; T1–T3 = pregnancy assessments; T4 = postpartum assessment.

Supplementary Table S1. Baseline Comparisons Between Included and Excluded Participants

Variable	Analytic sample n	Analytic sample M (SD)	Excluded participants n	Excluded M (SD)	Mean difference [95% CI]	Welch's t(df)	p	Cohen's d
Age at baseline	151	25.18 (3.80)	19	25.58 (3.99)	−0.40 [−2.40, 1.60]	−0.41 (22.3)	0.683	−0.10
Years of education	148	14.98 (2.05)	18	15.67 (1.97)	−0.69 [−1.71, 0.34]	−1.39 (21.7)	0.178	−0.34
Depressive symptoms, T0	150	4.09 (3.14)	17	4.35 (2.64)	−0.27 [−1.70, 1.17]	−0.39 (21.5)	0.704	−0.09
Anxiety symptoms, T0	150	2.93 (2.67)	17	2.82 (3.05)	0.11 [−1.50, 1.72]	0.14 (18.9)	0.888	0.04
Perceived social support, T0	151	81.40 (5.40)	16	80.00 (9.05)	1.40 [−3.48, 6.29]	0.61 (16.1)	0.551	0.19
Learned helplessness, T0	151	37.10 (8.51)	14	36.71 (6.33)	0.39 [−3.46, 4.23]	0.21 (17.7)	0.836	0.05

Note. Excluded participants are those who provided baseline data but were not included in the analytic sample. Mean differences and Cohen's d values are calculated as the analytic sample minus the excluded participants; positive values indicate higher scores in the analytic sample. Welch's t-tests were used because group sizes were unequal. Comparisons were based on available baseline data; sample sizes vary because not all excluded participants completed all baseline measures. T0 = preconception baseline.

Supplementary Table S2. Parallel Mediation Model of the Association Between Preconception LH and Postpartum Bonding (N = 151)

Effect	b	SE	95% CI	β	z	p
Indirect effects						
LH → Antenatal bonding → Postpartum bonding	−0.103	0.037	[−0.188, −0.042]	−0.101	−2.81	0.005
LH → Prenatal depressive symptoms → Postpartum bonding	−0.040	0.030	[−0.122, 0.016]	−0.039	−1.34	0.181
LH → Prenatal anxiety symptoms → Postpartum bonding	0.005	0.031	[−0.053, 0.073]	0.005	0.15	0.882
Component paths						
LH → Antenatal bonding	−0.211	0.061	[−0.327, −0.100]	−0.271	−3.46	<0.001
Antenatal bonding → Postpartum bonding	0.488	0.101	[0.293, 0.685]	0.371	4.83	<0.001
LH → Prenatal depressive symptoms	0.110	0.032	[0.055, 0.170]	0.272	3.47	<0.001
Prenatal depressive symptoms → Postpartum bonding	−0.368	0.254	[−0.960, 0.146]	−0.145	−1.45	0.147
LH → Prenatal anxiety symptoms	0.099	0.025	[0.046, 0.149]	0.304	3.92	<0.001
Prenatal anxiety symptoms → Postpartum bonding	0.047	0.314	[−0.524, 0.659]	0.015	0.15	0.882
Direct and total effects						
Direct effect: LH → Postpartum bonding	−0.324	0.078	[−0.507, −0.153]	−.316	−4.17	<0.001
Total effect: LH → Postpartum bonding	−0.475	0.078	[−0.657, −0.314]	−.463	−6.11	<0.001

Note. Antenatal bonding, prenatal depressive symptoms, and prenatal anxiety symptoms were indexed as mean scores across available T1–T3 assessments. b = unstandardized coefficient; SE = standard error; CI = 95% bootstrap percentile confidence interval based on 5,000 bootstrap samples; β = completely standardized coefficient; LH = learned helplessness.

Supplementary Text S1. Sensitivity and Follow-Up Analyses

Sensitivity analyses examined whether the LH-related findings remained robust after adjustment for additional baseline covariates. Specifically, the mixed-effects model for antenatal bonding, the hierarchical regression model for postpartum bonding, and the parallel mediation model were rerun with additional adjustment for age at baseline, years of education, baseline depressive and anxiety symptoms, and baseline perceived social support. In the adjusted antenatal bonding model, preconception LH remained a significant negative predictor of antenatal bonding, $b = -0.199$, $SE = 0.066$, 95% CI [−0.328, −0.067], $t(134) = -3.02$, $p = 0.003$. This model included 396 observations nested within 146 participants. In the adjusted postpartum bonding regression, adding preconception LH after baseline covariates and concurrent postpartum depressive and anxiety symptoms significantly improved model fit, $\Delta R^2 = 0.060$, $F(1, 120) = 12.7$, $p < 0.001$. Preconception LH remained a significant negative predictor of postpartum bonding, $b = -0.289$, $SE = 0.081$, 95% CI [−0.449, −0.128], $\beta = -.286$, $p < 0.001$. This model included 129 complete cases. The parallel mediation model was then rerun to test whether the indirect pathway through antenatal bonding remained robust after adjustment for the same baseline covariates. The adjusted model included 147 participants. The indirect effect through antenatal bonding remained significant, $b = -0.103$, $SE = 0.039$, 95% bootstrap CI [−0.185, −0.037], $\beta = -.101$, $p = 0.008$. By contrast, the indirect effects through prenatal depressive symptoms, $b = -0.025$, $SE = 0.022$, 95% bootstrap CI [−0.079, 0.011], $p = 0.254$, and prenatal anxiety symptoms, $b = 0.011$, $SE = 0.014$, 95% bootstrap CI [−0.018, 0.060], $p = 0.431$, remained nonsignificant. The direct effect of preconception LH on postpartum bonding also remained significant, $b = -0.285$, $SE = 0.082$, 95% CI [−0.472, −0.100], $\beta = -.278$, $p < 0.001$.

Additional checks evaluated whether postpartum assessment timing or obstetric and perinatal variables warranted inclusion as covariates. Time from childbirth to completion of the postpartum assessment was not associated with postpartum bonding, as indicated by both Pearson correlation, $r(131) = 0.01$, $p = 0.904$, and Spearman correlation, $\rho = -.006$, $p = 0.946$. Gestational age at birth and birthweight were not significantly correlated with antenatal or postpartum bonding, with all $|r| \leq 0.14$ and all $ps \geq 0.101$. An ANOVA model including categorical obstetric and perinatal variables (newborn sex, pregnancy course, mode of delivery, NICU admission, and prolonged maternal postpartum hospitalization) was nonsignificant, $F(7, 124) = 1.06$, $p = 0.391$. None of the individual categorical predictors was significant, with all $ps \geq 0.197$. Therefore, postpartum assessment timing and obstetric or perinatal variables did not warrant inclusion as additional covariates.

Finally, exploratory follow-up models tested whether the nonsignificant prenatal symptom effects in the antenatal bonding model reflected overlap with preconception LH or shared variance between depressive and anxiety symptoms. Full estimates from models excluding preconception LH are reported in Supplementary Table S3. When prenatal depressive and anxiety symptoms were examined simultaneously, neither within-person deviations nor between-person mean levels of either symptom domain were significantly associated with antenatal bonding. When symptom domains were examined separately, prenatal depressive symptoms showed negative but nonsignificant trends at both the within-person level, $b = -0.145$, $p = 0.097$, and the between-person level, $b = -0.301$, $p = 0.055$, whereas prenatal anxiety symptoms were not significantly associated with antenatal bonding. This pattern suggests that prenatal affective symptoms did not show robust associations with antenatal bonding in these follow-up models.

Supplementary Table S3. Follow-Up Mixed-Effects Models Examining Prenatal Affective Symptoms as Predictors of Antenatal Bonding After Excluding Preconception LH

Model	Predictor	b	SE	95% CI	t	df	p
**Model 1: Prenatal depression and anxiety simultaneously**	Intercept	71.270	0.602	[70.109, 72.428]	118.50	227	<0.001
	Time: T2 vs. T1	4.410	0.497	[3.427, 5.367]	8.88	256	<0.001
	Time: T3 vs. T1	7.877	0.507	[6.842, 8.861]	15.55	258	<0.001
	Prenatal depressive symptoms (WP)	−0.096	0.097	[−0.288, 0.089]	−1.00	251	0.320
	Prenatal anxiety symptoms (WP)	−0.142	0.123	[−0.379, 0.101]	−1.15	251	0.252
	Prenatal depressive symptoms (Mean)	−0.324	0.214	[−0.727, 0.088]	−1.51	154	0.133
	Prenatal anxiety symptoms (Mean)	0.041	0.265	[−0.464, 0.551]	0.16	150	0.876
**Model 2: Prenatal depression only**	Intercept	71.305	0.599	[70.122, 72.501]	119.11	228	<0.001
	Time: T2 vs. T1	4.335	0.492	[3.354, 5.293]	8.81	257	<0.001
	Time: T3 vs. T1	7.833	0.506	[6.834, 8.809]	15.50	259	<0.001
	Prenatal depressive symptoms (WP)	−0.145	0.087	[−0.315, 0.028]	−1.67	252	0.097
	Prenatal depressive symptoms (Mean)	−0.301	0.156	[−0.603, 0.001]	−1.93	151	0.055

**Model 3: Prenatal anxiety only**	Intercept	71.137	0.595	[70.032, 72.324]	119.54	218	<0.001
	Time: T2 vs. T1	4.583	0.469	[3.639, 5.514]	9.76	257	<0.001
	Time: T3 vs. T1	8.038	0.483	[7.084, 8.993]	16.65	258	<0.001
	Prenatal anxiety symptoms (WP)	0.037	0.224	[−0.405, 0.462]	0.17	243	0.869
	Prenatal anxiety symptoms (Mean)	−0.232	0.194	[−0.604, 0.153]	−1.20	147	0.233

Note. All models included 404 observations nested within 150 participants and a random intercept for participant. Antenatal bonding was assessed using MAAS total scores at T1–T3. Time was modeled as a categorical fixed effect, with T1 as the reference category. Continuous predictors were centered on the sample mean. WP = within-person deviations from each participant's mean symptom level across pregnancy; Mean = between-person mean symptom level across T1–T3. *b* = unstandardized coefficient; *SE* = standard error; *CI* = 95% bootstrap percentile confidence interval based on 5,000 resamples; *df* = degrees of freedom estimated using the Satterthwaite approximation; LH = learned helplessness. Model 1: marginal R^2 =0.192, conditional R^2 =0.768; Model 2: marginal R^2 =0.192, conditional R^2 =0.766; Model 3: marginal R^2 =0.182, conditional R^2 =0.767.
